# What is the research evidence for antibiotic resistance exposure and transmission to humans from the environment? A systematic map protocol

**DOI:** 10.1186/s13750-020-00197-6

**Published:** 2020-06-03

**Authors:** Isobel C. Stanton, Alison Bethel, Anne F. C. Leonard, William H. Gaze, Ruth Garside

**Affiliations:** 1 European Centre for Environment and Human Health, College of Medicine and Health, Penryn Campus, University of Exeter, Penryn, TR10 9FE UK; 2 College of Medicine and Health, St Luke’s Campus, University of Exeter, Exeter, EX1 1TX UK; 3 European Centre for Environment and Human Health, College of Medicine and Health, Knowledge Spa, University of Exeter, Truro, TR1 3HD UK

**Keywords:** Antibiotic resistance, Health, Colonisation, Infection, Water, Air, Soil, Faeces, Food

## Abstract

**Background:**

Antimicrobial resistance (AMR) is a public health crisis that is predicted to cause 10 million deaths per year by 2050. The environment has been implicated as a reservoir of AMR and is suggested to play a role in the dissemination of antibiotic resistance genes (ARGs). Currently, most research has focused on measuring concentrations of antibiotics and characterising the abundance and diversity of ARGs and antibiotic resistant bacteria (ARB) in the environment. To date, there has been limited empirical research on whether humans are exposed to this, and whether exposure can lead to measureable impacts on human health. Therefore, the objective of this work is to produce two linked systematic maps to investigate previous research on exposure and transmission of AMR to humans from the environment. The first map will investigate the available research relating to exposure and transmission of ARB/ARGs from the environment to humans on a global scale and the second will investigate the prevalence of ARB/ARGs in various environments in the UK. These two maps will be useful for policy makers and research funders to identify where there are significant gluts and gaps in the current research, and where more primary and synthesis research needs to be undertaken.

**Methods:**

Separate search strategies will be developed for the two maps. Searches will be run in 13 databases, and grey literature will be sought from key websites and engagement with experts. Hits will be managed in EndNote and screened in two stages (title/abstract then full text) against predefined inclusion criteria. A minimum of 10% will be double screened with ongoing consistency checking. All included studies will have data extracted into a bespoke form designed and piloted for each map. Data to be extracted will include bibliographic details, study design, location, exposure source, exposure route, health outcome (Map 1); and prevalence/percentage of ARB/ARG (Map 2). No validity appraisal will be undertaken. Results will be tabulated and presented narratively, together with graphics showing the types and areas of research that has been undertaken and heatmaps for key exposure-health outcomes (Map 1) and exposure-prevalence (Map 2).

## Background

Antibiotics are antimicrobials used to prevent and treat bacterial infections. Their efficacy is important as their use is critical in human and veterinary medicine, as well as in food production practices [1]. Presently, the efficacy of antibiotics is being undermined by the ability of bacteria to resist the actions of these drugs (antibiotic resistance). This results in treatment failure, prolonged morbidity and increased risk of mortality when treating infections caused by resistant pathogens [2]. The devastating impact of such resistance has led to antibiotic resistance and antimicrobial resistance (AMR) more broadly, being placed on the UK Risk Register, alongside other critical risks such as climate change, pandemic influenza and global terrorism [3, 4]. It has been predicted that by 2050 AMR will be the leading cause of death globally, accounting for 10 million deaths per year [2]. The term AMR includes resistance to agents with antimicrobial properties by microorganisms, such as bacterial, fungal, viral or parasitic organisms. It is frequently used interchangeably in the literature with the term antibiotic resistance. However, the focus of this study will be antibiotic resistant bacteria (ARB).

The occurrence of infections that are resistant to antimicrobials is increasing globally [1, 5, 6]. Successful treatment of resistant bacterial infections increasingly relies on more expensive “last-resort” antibiotics. Resistance has, however, already developed to such antibiotics, for example, colistin [7]. In addition, there are few novel antimicrobials being discovered and coming to market, as the investment (in time and money) needed to develop these drugs is high and as small returns is promised in the lifetime of the patent [8]. In the last three decades, only two antibiotic classes, oxazolidinones and cyclic lipopeptides, have been brought to market [9]. Without the discovery of new antibiotics, and with the development of resistance, it is possible that we will enter an era similar to that before the discovery of antibiotics, where routine medical procedures become far riskier as a result of our inability to prevent or treat infections which are currently simple to treat [10].

The 2014 O’Neill report, commissioned by the UK government, stated that the global number of deaths annually from AMR will increase from 700,000 in 2014 to 10 million by 2050. This report also predicted that AMR will negatively impact the global economy, estimating a drop in the world’s GDP of 2.5% to 3% (up to 100 trillion US dollars) between 2014 and 2050 as a consequence of AMR [2]. In the UK, the Chief Medical Officer stated that AMR infections already cost the NHS approximately £180 million every year [11].

There is a vast range of research that has been undertaken investigating the impact of AMR from a clinical perspective [12–17]. However, the role of the environment in the dissemination of AMR is only recently being investigated. Human use of antibiotics has been implicated in the rise of resistance, with our activities accelerating the development and dissemination of antibiotic resistance genes (ARGs) in and to the environment. In 2017, the United Nations Environment Programme Frontiers report highlighted AMR as the most critical emerging environmental pollution issue [18]. Although AMR is an ancient and natural phenomenon [19], the increased use of compounds with antimicrobial properties and their subsequent release into the environment through anthropogenic activities has increased the rate of the development of novel resistance and dissemination in such compartments [20]. For example, a large proportion (up to 90% [21]) of administered antibiotic is excreted in its biologically active form in both urine and faeces from treated patients and animals and this can be released into receiving environments such as soil and water [12] in concentrations that range from ng/L to µg/L [22]. Research has shown that these environmentally relevant concentrations of antibiotics are able to select for ARGs [23–27]. As a result of both naturally occurring ARGs, and selective pressure from antimicrobial compounds used by humans, research has found that ARGs are found ubiquitously throughout environmental compartments [20]. Furthermore, there is evidence that ARGs in environmental bacteria have the potential to be taken up by human-associated bacteria and pathogenic bacteria via horizontal gene transfer [28, 29], and that people exposed to AMR in the environment are at greater risk of being faecal carriers of AMR compared to people with less exposure [30].

**Fig. 1:**
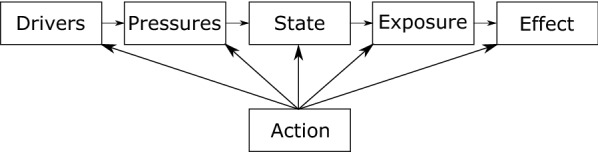
DPSEEA Framework adapted from Morris et al. [32]

This topic area has been previously mapped to the Driver-Pressure-State-Exposure-Effect-Action (DPSEEA) Framework [31]. The basic DPSEEA Framework can be seen in Fig. 1.

For environmental AMR research: “Driver” may refer to increasing number of resistant infection; “Pressure” may refer to inappropriate disposal of antimicrobials or other co-selecting agents into the environment; “State” may refer to the concentration levels of antimicrobials in the environment or abundance of resistant organisms or genes; “Exposure” may refer to direct contact with the environment resulting in inhalation or consumption of resistant organisms or genes; “Effect” may refer to the human health outcome of this exposure and “Action” may refer to any action taken at any stage of this framework to mitigate the human health outcome.

The majority of the research undertaken investigating AMR in the environment has focused on identifying and quantifying the discharge of various chemical and microbial pollutants (Pressure and State), including the concentrations of antimicrobials [33–35], ARB [36–38] and ARGs [39–41] in the environment. However, there is limited data on the fate of these pollutants (Exposure and Effect). The question of whether AMR levels in the environment are high enough to pose an exposure risk to humans, and if environmental resistant bacteria are transmitted to humans and threaten public health remains and is a key consideration for policy-makers around the world.

There has been relatively little evidence synthesis work on environmental AMR. One recent systematic review investigated what was the most effective control measure in preventing the dissemination of AMR in the environment [42]. This work, however, did not investigate the potential exposure and transmission of environmental AMR to humans. To the best of the authors’ knowledge, there has not been any evidence synthesis work investigating this.

The aim of this article is to provide a systematic map protocol. Here we are following ROSES for systematic map protocol reporting standards (provided as an Additional file 1) and CEE guidance for methods.

## Stakeholder engagement

Initial revising of the scope of the question for the two maps was undertaken through a meeting with a member from the Environment Agency.

Subsequently, a number of stakeholders were asked to make comments on the inclusion and exclusion criteria, search terms and sources of grey literature via email. These stakeholders were from: Animal and Plant Health Agency; AstraZeneca; Centre for Environment, Fisheries and Aquaculture Science; Department for Environmental, Food & Rural Affairs; Environment Agency; Food Standards Agency; Joint Nature Conservation Committee; Public Health England; Severn Trent Water and Veterinary Medicines Directorate.

A further meeting was held to further discuss sources of grey literature searching, outputs of the maps that stakeholders would find most useful and potential means of dissemination of the outputs through stakeholder organisations using directed questions. This was attended by members from organisations mentioned above, in addition to: Department of Agriculture, Environment and Rural Affairs; GlaxoSmithKline; Public Health England; Royal Devon & Exeter Hospital Trust; Severn Trent Water; Welsh Government and Wessex Water.

Finally, stakeholders will be consulted to discuss preliminary findings, to ensure that outputs are meaningful to specific organisations and that key gaps and gluts of evidence are prioritised for the maps. Outputs will not exclusively be those which are suggested by stakeholders and suggestions from stakeholders will be discussed by the team to ensure non-biased outputs whilst encompassing stakeholder suggestions and priorities. These discussions were originally planned to be undertaken as another formal meeting. As a result of the global pandemic this will now take place via informal means such as email, virtual meetings and/or telephone interviews.

## Objective of review

The aim is to conduct systematic mapping exercise in order to provide data on current evidence and identify research gluts and gaps relating to AMR in the environment and whether this can affect human health.

Primary research question: What research evidence is there that humans are exposed to and affected by AMR in the environment?

This will be broken down into two evidence maps:

Map 1: What research evidence is there about ARB exposure and transmission to humans from the environment?

This will include studies that investigate either measureable health outcomes (e.g. colonisation, infection or mortality) or an estimated exposure risk in humans via direct contact such as inhalation or consumption from antibiotic resistant bacteria from environmental sources (e.g. water or soil sources).

Map 2: What research evidence is there measuring the prevalence of ARB in the environment in the UK? This will be UK based as the funding’s remit is for UK policy based research. This will help to elucidate which environments have been extensively researched and which are currently under researched.

This will include studies that investigate the prevalence, percentage or abundance of resistant bacteria in environments such as various water environments and soil environments for example.

## Methods

### Searching for articles

Medline via OvidSP has been used to develop and refine the bibliographic database searches for both maps by the information specialist (AB) through a process of scoping, checking against a list of known includable papers and with the project team. Both free text terms and controlled vocabulary terms were used when available and relevant. This search will be translated across the following databases:

Medline (1946–present), CAB Abstracts (1973–present), Global Health (via OvidSP, 1973–present), BIOSIS Citation Index (1969–present), Web of Science Core Collection (via Web of Science, 1900–present), GreenFILE, Environment Complete (via EBSCOhost, 1888–present), SCOPUS (1788–present), Epistemonikos, ProQuest Dissertations and Theses Global (via ProQuest, 1637–present), Explore (via the British Library), and AGRIS (via FAO). Please note that these databases will be limited by a search date (details can be found below).

All the results for both maps will be downloaded into Endnote X8 where duplicates will be removed. Supplementary search methods will be used for both maps to attempt to capture additional relevant information including forwards and backwards citation searching of included articles, hand searching of relevant journals and searching within organisational websites.

The Medline search string for both maps can be found in an Additional file 2 (Medline search strategy).

For Map 2:

To focus searches to geographically relevant studies, a modified version of the MEDLINE Ovid UK search filter [43] will be used for all the bibliographic database searches in Map 2.

Key author searches on both Web of Science Core Collection and SCOPUS will be run for Gaze, W. Wellington, E. and Amos, GC. These authors were chosen based on expert advice as key people working and publishing in this field in the UK. These results will be added to the other database search results.

Google scholar searches will also be carried out using a simplified search string with the first 1000 hits being downloaded. We will search google scholar using the Publish or Perish software using the following two strings: “Antimicrobial resistance and UK” and “Antimicrobial resistance and United Kingdom” both just in the title. These results will be added to the other search results.

The following websites will be searched:

Cefas (Centre for Environment, Fisheries and Aquaculture Science)

EA (Environment Agency)

SEPA (Scottish Environmental Protection Agency)

Defra (Department of Environment, Food and Rural Affairs)

APHA (Animal and Plant Health Agency)

VMD (Veterinary Medicines Directorate)

PHE (Public Health England)

CARS (Control of Antimicrobial Resistance Scotland)

HPS (Health Protection Scotland)

Welsh government

### Limits

For both maps, only studies published in English will be considered due to limited resources and as this is relevant to UK policy scenarios for Map 2. Map 1 will be date limited to evidence published from 2009 and Map 2 date limited to 2005.

### Estimating the comprehensiveness of the search

The comprehensiveness of both of the maps have been tested by using key papers that were known to be included after full text screening to validate that the searches were identifying these. For Map 1, 11 publications were used and for Map 2, 8 publications were used. A list of these references for both maps can be found as an Additional file 3 (Key papers). The final searches were able to identify all key papers for both questions.

### Search update

The searches will not be updated prior to publication as this is a short term project. It is expected that the results of this systematic mapping exercise will be available within a year of the search date.

## Article screening and study eligibility criteria

### Screening strategy

Studies retrieved from the searches will be imported into EndNote, duplicates will be removed and articles will be screened in two stages. An initial screening of 100 articles will be undertaken at title and abstract stage and discussion of all will occur between reviewers to ensure consistency. Titles and abstracts will be screened against the inclusion and exclusion criteria by one reviewer (as a result of resource constraints), with at least 10% of results being screened by a second reviewer to ensure consistency. Kappa coefficient will be used to evaluate consistency between the two reviewers. The kappa coefficient will be used as a guide and if the score is equal to or below 0.6, this will prompt discussion points, refining of the eligibility criteria and a further evaluation of 100 articles will be undertaken and discussions will occur. In addition, all discrepancies will be discussed by the two reviewers and the wider review team will be consulted if necessary. At the second stage, full texts identified in the first screening will be assessed for their eligibility with one reviewer screening and extracting meta-data from all articles (as a result of resource constraints) and a minimum of 10% being screened and extracted by a second reviewer. Reviewers that have authored papers which are found during the searching process will not review these publications to avoid biases towards these publications. These papers will be screened at both abstract and title screening and full text screening by an impartial reviewer.

A list of studies that are excluded at full text screening will be made available with reasons for exclusion.

### Eligibility criteria

#### Map 1

Details of the eligibility criteria and justifications for these are presented in Table 1. The question is expressed in a PEO format (Population, Exposure, Outcome) with the exposure sources and routes both considered important for this question.

**Table 1:** Eligibility criteria for Map 1

	Inclusion	Exclusion	Justification
Population	Adults, children	Non-humans (e.g. animals, plants)	Evidence of ARB transmission to humans from the environment is of interest to relevant stakeholders
Exposure sources	Meat from wild animals, including shellfish (bivalve molluscs, lobster, crab, etc.), fin fish, game; plants that are consumed raw (including salad, fruit etc.)	Meat and animal products from commercially produced animals including fish, shellfish (including shrimp), poultry (including pheasants), pigs, cows, sheep etc., and products including honey, milk, eggs; Plants that are always consumed cooked (including grains etc.)	While food is produced in the environment, practices during commercial production and processing (e.g. antibiotic use, through poor hygiene in preparation and handling of food) might be the sources of ARB, rather than from the environment Wild animals consumed for their meat are of interest as sources of AMR are more likely to be from the environment. Bivalve molluscs are grown in the environment and are filter-feeders, concentrating contaminants in the environment, and this meat is typically eaten raw or lightly cooked. Shrimp are intensively raised in some parts of the world, and antibiotic usage is poorly regulated. Likewise, pheasants are reared on high levels of antibiotics and then released into the environment [46] Plants consumed raw pose are more likely to result in transmission of ARB from plants to humans
	Water in the environment (including water used in crop irrigation; aquaculture; ambient surface waters used for recreation; drinking water and wastewater from domestic and industrial sources)	Water from chlorinated swimming pools and spas	Water in swimming pools and spas are treated to remove pathogenic microorganisms, and are not considered the environment
	Soil including that conditioned with faecal matter (sludge/slurry/manure etc. or irrigated with waste water). Exposure may be through activities such as farming, gardening, leisure activities such as playing etc.)	N/A	
	Outdoor air (may contain dust, water droplets etc.)	Studies that have collected air from indoor environments	ARB in outdoor environment are more likely to be from natural sources
	Contact with animals or their faeces	Pets, companion animals and commercially produced livestock	ARB in/on wild animals are more likely to be from the environment. Those on pets, companion animals and commercially produced animals might be due to antimicrobial usage during animal rearing, for example raw food diet in companion animals is associated with carriage of AMR bacteria [47]. Exposure and transmission from pets, companion animals and commercially produced livestock are not of specific interest to relevant stakeholders
Exposure routes	Consumption/ingestion; Inhalation; Direct contact		
Outcomes	Mortality caused by infection with ARB or bacteria harbouring ARG(s); Infection with ARB or bacteria harbouring ARG(s); Colonisation by ARB or bacteria harbouring ARG(s); Estimated or measured risk of exposure to ARB or bacteria harbouring ARG(s)	Infections caused by fungi, parasites or viruses	While fungal, parasitic and viral infections resistant to antimicrobials are of interest to relevant stakeholders, ARB are a priority for regulators Resource constraints mean other types of AMR organisms will not be included

*ARB* antibiotic resistant bacteria, *ARG* antibiotic resistance gene, *AMR* antimicrobial resistance

#### Map 2

Details of the eligibility criteria justifications for these are presented in Table 2. The question is expressed in a PEO format (Population, Exposure, Outcome) with the exposure sources and routes both considered important for this question.

**Table 2:** Eligibility criteria for Map 2

	Inclusion	Exclusion	Justification
Population	Bacteria	Fungi, parasites, viruses	ARBs are a priority interest for relevant stakeholders. Resource constraints mean other types of AMR organisms will not be included
Exposure sources	As in Table 1	As in Table 1	As in Table 1
Exposure routes	Exposure to AMR organisms		
Outcomes	Prevalence/percentage of ARB Prevalence/percentage of ARGs	Presence of ARB/ARGs with no quantification	

*ARB* antibiotic resistant bacteria, *ARG* antibiotic resistance gene, *AMR* antimicrobial resistance

### Relevant types of study

Studies will be commercially published work and grey literature. For Map 1, these studies will include systematic reviews, experimental studies (randomised exposure trials), observational studies (prospective/retrospective cohort studies, cross-sectional studies, case studies and case series) and modelling studies (for example quantitative microbial risk assessments). For Map 2, relevant study types will include systematic reviews and environmental surveillance studies.

### Location

Whilst Map 1 will not include geographic limitations on study location, Map 2 will be restricted to only include studies where data is available from locations in the United Kingdom. There are expected to be many more eligible studies for Map 2 than for Map 1, and due to limited resources, data pertinent to the UK has been prioritised so that this map will be of most use to UK based policy makers and stakeholders.

### Date range

For Map 1, literature published in the past 10 years (from 2009) will be included. Older studies will be excluded, but eligible research prior to this date is unlikely. For Map 2, searching will be extended to include studies published between 2005 and the present, as this is when interest in the topic of AMR in the environment started to increase. These dates have been selected based on advice from experts in the field.

### Study validity assessment

No formal validity appraisal of included studies will be performed. However, study designs of included studies will be extracted for the maps.

### Data coding strategy

For both of the maps we will extract the following meta-data: bibliographic information; study location; study type; exposure source (main category—water, food, air, sediment, soil, animal faeces); exposure source (sub-categories. For example—Water: coastal, river, lake, groundwater, wastewater treatment plant influent, wastewater treatment plant effluent, drinking, crop irrigation etc.); whether there was a comparator and outcome.

For Map 1, information on population types and exposure route will also be extracted.

For Map 2, information about the experimental methodology used will also be extracted.

### Study mapping and presentation

We will follow ROSES guidance for reporting on the systematic maps [44]. As well as the narrative synthesis of the systematic maps, data collected from the eligible studies will be recorded in an Excel spreadsheet and made available as an additional file. Both maps will be presented using the EviAtlas tool and will be uploaded online [45]. We plan to display: the location of the studies; heatmaps showing different exposure sources or routes by different outcome measures and details of the impact to relevant populations.

## Supplementary information

**Additional file 1.** ROSES for Systematic Map Protocols checklist.

**Additional file 2.** Medline search strategy.

**Additional file 3.** Key papers for validating the search strategy.

## Data Availability

Not applicable
