# Supplementary material for: What is the research evidence for antibiotic resistance exposure and transmission to humans from the environment? A systematic map protocol
Source: Environ Evid. 2020 Jun 3;9(1):12. doi: 10.1186/s13750-020-00197-6 (PMC7268584; doi:10.1186/s13750-020-00197-6)
Supplement: Supplementary file 2 — Additional file 2. Medline search strategy. [file 13750_2020_197_MOESM2_ESM.docx]

**Map 1:**

**Medline search strategies**

1. amr.tw.

2. anti biotic*.tw.

3. antibiotic*.tw.

4 Enterobacteria*.tw.

5 antimicrobial*.tw.

6 microbial*.tw.

7 antibacter*.tw.

8 Macrolide*.tw.

9 betalactam*.tw.

10 Beta lactam*.tw.

11 Bacter*.tw.

12 drug*.tw.

13 multidrug*.tw.

14 Resistan*.tw.

15 Coresist*.tw.

16 Select*.tw.

17 Coselect*.tw.

18 Resistome*.tw.

19 Mobilome*.tw.

20 select.tw.

21 selection.tw.

22 produce.tw.

23 producing.tw.

24 14 or 15 or 17 or 18 or 19 or 20 or 21 or 22 or 23

25 5 or 7

26 14 and 25

27 2 or 3 or 4 or 6 or 8 or 9 or 10 or 11 or 12 or 13

28 ((anti biotic* or antibiotic* or Enterobacteria* or microbial* or Macrolide* or betalactam* or Beta lactam* or Bacter* or drug* or multidrug*) adj1 (Resistan* or Coresist* or Coselect* or Resistome* or Mobilome* or select or selection or produce or producing)).tw.

29 1 or 26 or 28

30 15 or 16 or 17 or 18 or 19 or 22 or 23

31 ((amr or antimicrobial* or antibacter*) adj1 (Coresist* or Select* or Coselect* or Resistome* or Mobilome* or produce or producing)).tw.

32 29 or 31

33 human*.tw.

34 public*.tw.

35 patient*.tw.

36 clinical*.tw.

37 33 or 34 or 35 or 36

38 airborne.tw.

39 Food*.tw.

40 water*.tw.

41 soil*.tw.

42 wildlife*.tw.

43 wastewater*.tw.

44 effluent*.tw.

45 wetland*.tw.

46 environment*.tw.

47 ocean*.tw.

48 sea.tw.

49 seas.tw.

50 aquatic*.tw.

51 river*.tw.

52 natural.tw.

53 stream*.tw.

54 sewage*.tw.

55 influent*.tw.

56 estuar*.tw.

57 pond*.tw.

58 38 or 39 or 40 or 41 or 42 or 43 or 44 or 45 or 46 or 47 or 48 or 49 or 50 or 51 or 52 or 53 or 54 or 55 or 56 or 57

59 amr.ti.

60 anti biotic*.ti.

61 antibiotic*.ti.

62 Enterobacteria*.ti.

63 antimicrobial*.ti.

64 microbial*.ti.

65 antibacter*.ti.

66 Macrolide*.ti.

67 betalactam*.ti.

68 Beta lactam*.ti.

69 Bacter*.ti.

70 drug*.ti.

71 multidrug*.ti.

72 Resistan*.ti.

73 Coresist*.ti.

74 Select*.ti.

75 produc*.ti.

76 59 or 60 or 61 or 62 or 63 or 64 or 65 or 66 or 67 or 68 or 69 or 70 or 71 or 72 or 73 or 74 or 75

77 Expos*.tw.

78 Transmi*.tw.

79 inhal*.tw.

80 consum*.tw.

81 contact*.tw.

82 transfer*.tw.

83 Infect*.tw.

84 impact*.tw.

85 health*.tw.

86 Comment/

87 letter/

88 editorial/

89 exp animals/ not humans.sh.

90 86 or 87 or 88 or 89

91 72 or 73 or 74 or 75

92 ((human* or public* or patient* or clinical*) adj4 (Expos* or Transmi* or inhal* or consum* or contact* or transfer* or Infect* or impact* or health*)).tw.

93 exp Drug Resistance, Bacterial/

94 32 or 93

95 58 and 76 and 92 and 94

96 95 not 90

97 96

98 limit 97 to yr="2009 -Current"

**Map 2:**

**Medline search strategies**

1. amr.tw
2. anti biotic*.tw.
3. antibiotic*.tw.
4. Enterobacteria*.tw.
5. entero bacteria*.tw.
6. antimicrobial*.tw.
7. microbial*.tw.
8. antibacter*.tw.
9. anti bacter*.tw.
10. Macrolide*.tw.
11. betalactam*.tw.
12. beta lactam*.tw.
13. resist*.tw.
14. coresist*.tw.
15. select*.tw.
16. resistome*.tw.
17. coselect*.tw.
18. mobilome*.tw.
19. 13 or 14 or 15 or 16 or 17 or 18
20. ((resist* or coresist* or select* or resistome* or coselect* or mobilome*) adj2 (anti biotic* or antibiotic* or Enterobacteria* or entero bacteria* or antimicrobial* or microbial* or antibacter* or anti bacter* or Macrolide* or betalactam* or beta lactam*)).tw.
21. drug resistance, microbial/ or exp drug resistance, bacterial/
22. exp United Kingdom/
23. ("national health service" or nhs).ti,ab,in.
24. (gb or "g.b." or britain or (british not "british columbia") or uk or "u.k." or united kingdom* or (england not "new england") or northern ireland* or nothern irish* or scotland* or scottish* or ((wales or "south wales") not "new south wales") or welsh*).ti,ab,jw,in.
25. 22 or 23 or 24
26. water*.tw.
27. soil*.tw.
28. wastewater*.tw.
29. air*.tw.
30. effluent*.tw.
31. wetland*.tw.
32. aquatic*.tw.
33. river*.tw.
34. sediment*.tw.
35. lake*.tw.
36. sea*.tw.
37. sewage*.tw.
38. influent*.tw.
39. environment*.tw.
40. natur*.tw.
41. exp Environmental Microbiology/
42. 26 or 27 or 28 or 29 or 30 or 31 or 32 or 33 or 34 or 35 or 36 or 37 or 38 or 39 or 40 or 41
43. 1 or 20 or 2
44. 25 and 42 and 43
45. limit 44 to yr="2005 -Current"
