# Supplementary material for: What is the research evidence for antibiotic resistance exposure and transmission to humans from the environment? A systematic map protocol
Source: Environ Evid. 2020 Jun 3;9(1):12. doi: 10.1186/s13750-020-00197-6 (PMC7268584; doi:10.1186/s13750-020-00197-6)
Supplement: Supplementary file 3 — Additional file 3. Key papers for validating the search strategy. [file 13750_2020_197_MOESM3_ESM.docx]

**Key papers**

**Map 1:**

1.         Chen MM, Boardman WS, Smith I, Goodman AE, Brown MH. Characterisation of beta-lactam resistance mediated by blaZ in staphylococci recovered from captive and free-ranging wallabies. J Glob Antimicrob Resist. 2015;3(3):184-9.

2.         Zurfluh K, Nüesch-Inderbinen MT, Poirel L, Nordmann P, Hächler H, Stephan R. Emergence of Escherichia coli producing OXA-48 β-lactamase in the community in Switzerland. Antimicrobial Resistance and Infection Control. 2015;4(1):9.

3.         Leonard AFC, Zhang L, Balfour AJ, Garside R, Hawkey PM, Murray AK, et al. Exposure to and colonisation by antibiotic-resistant E. coli in UK coastal water users: Environmental surveillance, exposure assessment, and epidemiological study (Beach Bum Survey). Environ Int. 2018;114:326-33.

4.         Varela AR, Manageiro V, Ferreira E, Guimarães MA, da Costa PM, Caniça M, et al. Molecular evidence of the close relatedness of clinical, gull and wastewater isolates of quinolone-resistant Escherichia coli. Journal of Global Antimicrobial Resistance. 2015;3(4):286-9.

5.         Lorenzin G, Piccinelli G, Carlassara L, Scolari F, Caccuri F, Caruso A, et al. Myroides odoratimimus urinary tract infection in an immunocompromised patient: an emerging multidrug-resistant micro-organism. Antimicrobial Resistance & Infection Control. 2018;7(1):96.

6.         Hatosy SM, Martiny AC. The Ocean as a Global Reservoir of Antibiotic Resistance Genes. Applied and Environmental Microbiology. 2015;81(21):7593-9.

7.         Abdolmaleki Z, Mashak Z, Safarpoor Dehkordi F. Phenotypic and genotypic characterization of antibiotic resistance in the methicillin-resistant Staphylococcus aureus strains isolated from hospital cockroaches. Antimicrob Resist Infect Control. 2019;8(1):54.

8.         Gomi R, Matsuda T, Matsumura Y, Yamamoto M, Tanaka M, Ichiyama S, et al. Whole-Genome Analysis of Antimicrobial-Resistant and Extraintestinal Pathogenic Escherichia coli in River Water. Appl Environ Microbiol. 2017;83(5).

**Map 2:**

1.         Sanganyado E, Gwenzi W. Antibiotic resistance in drinking water systems: Occurrence, removal, and human health risks. Sci Total Environ. 2019;669:785-97.

2.         Titilawo Y, Obi L, Okoh A. Antimicrobial resistance determinants of Escherichia coli isolates recovered from some rivers in Osun State, South-Western Nigeria: Implications for public health. Sci Total Environ. 2015;523:82-94.

3.         Carroll D, Wang J, Fanning S, McMahon BJ. Antimicrobial Resistance in Wildlife: Implications for Public Health. Zoonoses Public Health. 2015;62(7):534-42.

4.         Lopatek M, Wieczorek K, Osek J. Antimicrobial Resistance, Virulence Factors, and Genetic Profiles of Vibrio parahaemolyticus from Seafood. Applied and Environmental Microbiology. 2018;84(16).

5.         Adefisoye MA, Okoh AI. Ecological and Public Health Implications of the Discharge of Multidrug-Resistant Bacteria and Physicochemical Contaminants from Treated Wastewater Effluents in the Eastern Cape, South Africa. Water. 2017;9(8):18.

6.         Radhouani H, Igrejas G, Pinto L, Goncalves A, Coelho C, Rodrigues J, et al. Molecular characterization of antibiotic resistance in enterococci recovered from seagulls (Larus cachinnans) representing an environmental health problem. J Environ Monit. 2011;13(8):2227-33.

7.         Maloo A, Fulke AB, Mulani N, Sukumaran S, Ram A. Pathogenic multiple antimicrobial resistant Escherichia coli serotypes in recreational waters of Mumbai, India: a potential public health risk. Environ Sci Pollut Res. 2017;24(12):11504-17.

8.         Singh B, Tyagi A, Thammegowda NKB, Ansal MD. Prevalence and antimicrobial resistance of vibrios of human health significance in inland saline aquaculture areas. Aquac Res. 2018;49(6):2166-74.

9.         Xu Z, Shah HN, Misra R, Chen J, Zhang W, Liu Y, et al. The prevalence, antibiotic resistance and mecA characterization of coagulase negative staphylococci recovered from non-healthcare settings in London, UK. Antimicrobial Resistance & Infection Control. 2018;7(1):73.

10.       Petit F. Spread of antibiotic resistance in water: a public health and environmental issue. Environ Risque Sante. 2018;17:40-6.

11.       Hsu TTD, Mitsch WJ, Martin JF, Lee J. Towards sustainable protection of public health: The role of an urban wetland as a frontline safeguard of pathogen and antibiotic resistance spread. Ecol Eng. 2017;108:547-55.
